# Sulforaphane Attenuates Isoproterenol-Induced Myocardial Injury in Mice

**DOI:** 10.1155/2020/3610285

**Published:** 2020-12-19

**Authors:** Lijuan Song, Mudduluri Srilakshmi, Yi Wu, T. S. Mohamed Saleem

**Affiliations:** ^1^Department of Cardiology, The Second People's Hospital of Yunnan Province, Kunming, Yunnan 650021, China; ^2^Annamacharya College of Pharmacy, Rajampet-516126, Andhra Pradesh, India; ^3^Department of Cardiology, Zunyi Medical University, Guiyang, Guizhou 550001, China; ^4^Department of Pharmacology, College of Pharmacy, Riyadh ELM University, P.O. Box 84891, Riyadh 11681, Saudi Arabia

## Abstract

The development of isoproterenol- (ISO-) induced oxidative stress in the myocardium results in myocardial necrosis. Sulforaphane (SFN-0.4% of sulforaphane from standardized broccoli sprout extract) possesses chemoprotective, antidiabetic, and antibacterial activities and is also active against cardiovascular-related problems due to its antioxidant properties. This study was designed to investigate the cardioprotective effect of SFN against isoproterenol-induced myocardial injury in mice. Healthy male Swiss albino mice weighing 20–30 g were used in this study. These mice were randomly divided into five groups (*n* = 6). All the mice in the experimental groups received isoproterenol (5 mg/kg bw, via i.p.) consecutively for 2 days. The mice were treated with SFN (4 mg/kg bw) and *α*-tocopherol (TCF) (10 mg/kg bw) by oral gavage for 1-7 days as pre- and posttreatment for the prophylactic and treatment groups, respectively. On day 10, the following parameters were studied: heart weight to body weight ratio, antioxidant parameters, and cardiac markers; and mitochondrial enzymes were estimated for cardioprotection. Administration of isoproterenol in mice showed an increased level of serum cardiac markers and heart mitochondrial ATPase enzymes. An increased level of myocardial thiobarbituric acid-reactive substance and decreased levels of endogenous antioxidant enzymes indicated that oxidative stress is induced by isoproterenol in the myocardium. The administration of SFN in mice restored the levels of all biochemical parameters to near-normal levels. Histopathological studies further confirmed the protective effect of sulforaphane. This study concluded that treatment with SFN boosts the endogenous antioxidant activity and prevents isoproterenol-induced myocardial injury.

## 1. Introduction

Ischemic heart disease (IHD) is one of the leading causes of death with high mortality and morbidity rates worldwide [1]. Patients with IHD are prone to myocardial injury, which leads to myocardial dysfunction and myocardial ischemia [2]. Although several mechanisms were documented in the pathogenesis of IHD, excessive production of reactive oxygen species (ROS) was considered a prominent mechanism during myocardial ischemia [3, 4]. Therefore, reducing ROS during myocardial ischemic injury was considered the best treatment option [4]. Isoproterenol (ISO) is a synthetic -adrenergic agonist that leads to oxidative stress, causing significant myocardial injury. Upon oxidation, ISO produces quinone which generates a number of free radicals that cause oxidative and necrotic damage in the myocardium [5]. Although treatment using modern medicine was very effective, it was associated with more side effects. Therefore, researchers have been focusing on drug discovery from natural products. Moreover, plant-based drugs are economical and also cause lesser side effects [3].

**Figure 1:**
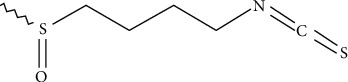


Sulforaphane (SFN) Figure 1 is a biologically active phytochemical present in many plants including some vegetables like cauliflower, broccoli, and cabbage. SFN is beneficial in reducing the risk of breast, bladder, and prostate cancers [6].

Several epidemiological studies have indicated that consumption of broccoli has a positive impact on health, many of them arbitrated by isothiocyanate SFN [7].

The development of oxidative stress through generation of free radicals has established pathogenesis mechanism for myocardial injury [3, 4]. The antioxidant potential of sulforaphane was well established and documented through several research [8, 9]. Hence, this study was carried out to determine the potential protective role of SFN through antioxidant and other protective mechanism against ISO-induced myocardial injury in mice as an animal model.

## 2. Materials and Methods

### 2.1. Drugs and Chemicals

SFN (from standardized broccoli sprout extract (0.4% of sulforaphane)) was obtained as a gift sample from Lebepur, Germany, and all chemicals purchased from Sigma-Aldrich, India, were of analytical grade.

### 2.2. Animals

Healthy male Swiss albino mice weighing between 20 and 30 g were used for the study. The animals were kept in sanitized polypropylene cages and in sterile paddy husk bedding in an air-conditioned room and were allowed access to a pellet diet and water ad libitum (Sainath Agency, Hyderabad). The mice were maintained at a temperature of 22 ± 2°C, relative humidity of 50 ± 5%, and 12 : 12 h light : dark cycle. All the mice were acclimatized to the laboratory conditions for 7 days before the actual study. The animals were divided into experimental and control groups. All the studies conducted were approved by the Institutional Animal Ethics Committee (IAEC/ANCP/2018-19/15).

### 2.3. Induction of Isoproterenol-Induced Myocardial Injury in Mice [10]

After a week of acclimatization, the animals were randomly divided into 5 groups with 6 mice in each group.

*Group 1*. Served as normal control and the mice received distilled water (10 ml/kg) through oral route.

*Group 2*. The mice received isoproterenol (ISO) 5 mg/kg bw, through i.p. for 2 consecutive days, and served as the toxic group.

*Group 3*. The mice received SFN 4 mg/kg bw, by oral gavage for 1–7 days, and served as the prophylactic group.

*Group 4*. The mice were treated with SFN 4 mg/kg bw, by oral gavage from days 3 up to 9, and served as the treatment group.

*Group 5*. The mice were treated with *α*-tocopherol (TCF) 10 mg/kg bw, by oral gavage from days 1 to 7, and served as the reference standard group.

Groups 3 and 5 received ISO 5 mg/kg bw, through i.p. for 2 consecutive days from day 8, and group 4 received ISO 5 mg/kg bw, through i.p. for 2 consecutive days from day 1.

### 2.4. Determination of Heart Weight/Body Weight Ratio

On day 10, the rats were killed by an overdose of pentobarbital (60 mg/kg bw i.p.) and the hearts were removed and weighed [11]. The wet heart weight to body weight ratio was calculated for evaluating the degree of myocardial weight gain. The heart weight/body weight ratio (mg/g) was calculated by dividing the heart weight by body weight.

### 2.5. Estimation of Biochemical Parameters in the Heart Homogenate

Heart tissues (0.5 g tissue) from mice were homogenized in 0.1 mM phosphate buffer, and the homogenate was used to estimate various biochemical parameters as follows.

#### 2.5.1. Estimation of Myocardial Thiobarbituric Acid-Reactive Substances (TBARS)

TBARS levels in the myocardium were measured using the method described by Okhawa et al. [12]. Briefly, 0.2 ml of homogenate was pipetted out, followed by the addition of 0.2 ml of 8.1% sodium dodecyl sulfate (SDS), 1.5 ml of 20% acetic acid (pH 3.5), and 1.5 ml of 0.8% thiobarbituric acid (TBA). Boiling of the tubes was done for 60 min at 95°C and then was cooled on ice. In the tubes, double distilled water (1.0 ml) and 5.0 ml of n-butanol–pyridine (15 : 1 *v*/*v*) mixture were added and centrifuged at 4000 × g for 10 min. The absorbance of the colour which was developed in the organic layer was measured at 532 nm. Data are expressed as nanomoles of TBARS/gram wet weight.

#### 2.5.2. Myocardial Reduced Glutathione (GSH)

Myocardial reduced glutathione (GSH) was estimated by the method of Ellman [13]. Briefly, the reaction mixture contains 0.1 ml of supernatant, 2.0 ml of 0.3 M phosphate buffer (pH-8.4), 0.4 ml of double-distilled water, and 0.5 ml of 5,5 dithiobis 2-nitrobenzoic acid (DTNB). Incubation of reaction mixture was done for 10 minutes, and then, absorbance was measured at 412 nm. Data are expressed as mole per gram wet weight.

#### 2.5.3. Myocardial Superoxide Dismutase (SOD)

Superoxide dismutase (SOD) levels in the hearts were determined by the modified method described by Kakkar et al. [14]. Briefly, the homogenate (0.6ml) was added to sodium pyrophosphate buffer (pH—8.3), followed by the addition of 0.1ml of 186M phenazine methosulfate, 0.3ml of 300 mM nitroblue tetrazolium, and 0.2ml of 780 M NADH. For 90 seconds, the reaction mixture was incubated at 30°C and then, the reaction was stopped by adding 1.0 ml of acetic acid, further 4.0 ml of n-butanol was added, and then, the reaction mixture was centrifuged at 3000 × g for 10 min. The absorbance of the organic layer was measured at 560 nm. Data are expressed as units per milligram protein.

#### 2.5.4. Myocardial Catalase

Catalase was estimated by the method described by Aebi [15]. Briefly, homogenate was added to a 3.0 ml cuvette containing 1.95 ml of 50 mM phosphate buffer (pH 7.0). Then, after adding1.0 ml of 30 mM hydrogen peroxide, changes in absorbance were followed for 30 s at 240 nm at an interval of 15 s. Catalase levels are expressed as units per milligram protein.

#### 2.5.5. Estimation of Glutathione Peroxidase (GPx)

Glutathione peroxidase was measured by the method described by Rotruck et al. [16]. Briefly, to 0.2 ml of tris buffer, 0.2 ml of EDTA, 0.1 ml of sodium azide, and 0.5 ml of tissue homogenate were added. To this mixture, 0.2 ml of glutathione and 0.1 ml of hydrogen peroxide were added. The contents were mixed well and incubated at 37°C for 10 minutes along with a tube containing all the reagents except sample. After 10 minutes, the reaction was arrested with the addition of 0.5 ml of 10% TCA and centrifuged and the supernatant was assayed for glutathione by Ellman's method. To 2.0 ml of the supernatant, 3.0 ml disodium hydrogen phosphate solution and 1.0 ml of DTNB reagent were added. The colour developed was read at 412 nm.

#### 2.5.6. Estimation of Protein

Protein estimation for the tissue sample of SOD and CAT was done by the method of Bradford [17]. A sample was added up to 20 *μ*l with double-distilled water, 50 *μ*l in sodium hydroxide, and 1 ml of Bradford reagent and kept aside for 10 min after vortexing. The absorbance was measured at 595 nm.

### 2.6. Estimation of Cardiac ATPase Activity

Na^+^/K^+^ ATPase activity, Mg^2+^ ATPase activity, and Ca^2+^ ATPase activity were estimated as described previously by Punithavathi and Prince [18]. Briefly, the mixture of 0.2 ml of heart homogenate, 1 ml of buffer, 0.2 ml of MgSO4, 0.2 ml of NaCl, 0.2 ml KCl, 0.2 ml of EDTA, and 0.2 ml of ATP were incubated at 37°C for 15 min. After 15 min, 1 ml of 10% TCA (ice-cold) was added to stop the reaction. From this, 1 ml supernatant was mixed with 4 ml of distilled water, and in this, 1 ml of 2.5% ammonium molybdate was added. This mixture was incubated at RT for 10 min, and finally, 0.4 ml of amino napthol sulfonic acid was added. OD was measured at 640 nm after 20 min by spectrophotometry.

### 2.7. Estimation of Cardiac Biomarkers

Lactate dehydrogenase (LDH) and creatine kinase (CK) levels in the serum were estimated by using respective kits as per the manufacturer's instruction booklet (Transasia Bio-Medicals Limited, Solan).

### 2.8. Histological Examination

Once the mice were sacrificed, the heart tissues were removed, washed immediately with saline, and then fixed in 10% buffered formalin. After fixation, the heart tissues were embedded in paraffin and these paraffin blocks were then cut into 5 *μ*m thick sections. These sections were stained with hematoxylin-eosin and then examined under the light microscope for histological changes.

### 2.9. Statistical Analysis

All values are expressed as mean ± standard error of the mean (SEM). All the data obtained for various biochemical parameters were analyzed using one-way analysis of variance (ANOVA) followed by Dunnett's multiple comparison test (GraphPad Version 5.0, La Jolla, CA, USA). *p* < 0.05 was considered statistically significant.

## 3. Result

### 3.1. Effect of Sulforaphane on Heart Weight/Body Weight Ratio

**Figure 2:**
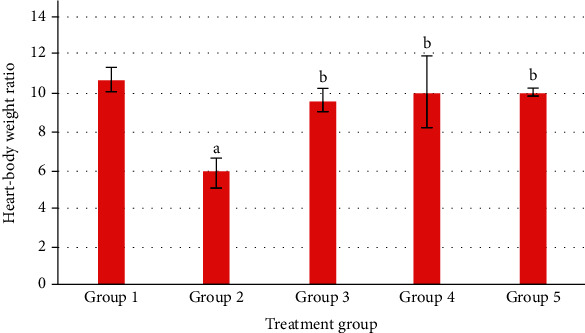
Effect of sulforaphane on heart and body weight ratio (mg/g) (a: *p* < 0.001*vs.* Group 1; b: *p* < 0.05*vs.* Group 2).

After the administration of ISO in mice, the heart weight/body weight ratio was found to be 5.883 ± 0.8195 mg/g, which was significantly (*p* < 0.01) low compared to the mice in the control group (10.68 ± 0.6204 mg/g). A significant increase in the heart weight to body weight ratio was observed in mice treated with SFN, in both the prophylactic (9.607 ± 0.5696 mg/g) and treatment groups (10.07 ± 1.911 mg/g) and also in the *α*-TCF- (10.10 ± 0.2049 mg/g) treated group. The results are presented in Figure 2.

### 3.2. Effect of Sulforaphane on Myocardial Thiobarbituric Acid-Reactive Substances (TBARS)

Mice administered with ISO showed high (*p* < 0.0001) levels of myocardial TBARS (345.0 ± 41.81 nmol/g wet wt) compared to the mice belonging to the control group (55.01 ± 10.59 nmol/g wet wt). A significant decrease in the TBARS levels was observed in mice treated with SFN, in both the prophylactic (147.1 ± 16.34 nmol/g wet wt) and treatment groups (166.5 ± 11.52 nmol/g wet wt) and also in the *α*-TCF (153.0 ± 5.61 nmol/g wet wt) treated group (Table 1).

### 3.3. Effect of Sulforaphane on Myocardial Glutathione (GSH)

A significant (*p* < 0.0001) decrease in the GSH level (19.89 ± 1.702 *μ*g/g wet wt) was observed in mice administered with ISO, compared to the control group (124.7 ± 2.442 *μ*g/g wet wt). A significant increase in the GSH level was observed in mice treated with SFN, in both the prophylactic (125.1 ± 2.175 *μ*g/g wet wt) and treatment groups (223.7 ± 1.635 *μ*g/g wet wt) and also in the *α*-TCF- (224.0 ± 2.216 *μ*g/g wet wt) treated group (Table 1).

### 3.4. Effect of Sulforaphane on Myocardial Superoxide Dismutase (SOD)

Mice administered with ISO showed a low level of SOD (13.70 ± 0.4703 IU/mg protein) compared to mice in the control group (39.13 ± 5.507 IU/mg protein). A significant increase in the levels of SOD was observed in mice treated with SFN in both the prophylactic (55.69 ± 5.988 IU/mg protein) and treatment groups (57.00 ± 3.505 IU/mg protein) and also in the *α*-TCF- (58.53 ± 2.397 IU/mg protein) treated group (Table 1).

### 3.5. Effect of Sulforaphane on Myocardial Catalase (CAT)

A significant decrease in the level of CAT (87.95 ± 3.591 IU/mg protein) was observed in mice administered with ISO compared to the mice in the control group (270.4 ± 8.789 IU/mg protein). A significant increase in the levels of CAT was observed in mice treated with SFN, in both the prophylactic (308.5 ± 52.24 IU/mg protein) and treatment groups (419.8 ± 91.55 IU/mg protein) and also in the *α*-TCF- (284.5 ± 27.49 IU/mg protein) treated group (Table 1).

### 3.6. Effect of Sulforaphane on Myocardial Glutathione Peroxidase (GPx)

Mice administered with ISO showed a low (*p* < 0.0001) level of GPx (5.766 ± 0.4945 *μ*g/g wet wt) compared to those in the control group (17.41 ± 0.5711 *μ*g/g wet wt). A significant increase in the GPx level was observed in mice treated with SFN, in both the prophylactic (8.602 ± 0.3663 *μ*g/g wet wt) and treatment groups (8.873 ± 0.4948 *μ*g/g wet wt) and also in the *α*-TCF- (10.94 ± 0.7333 *μ*g/g wet wt) treated group (Table 1).

### 3.7. Effect of Sulforaphane on Mitochondrial Enzymes (Table 2)

#### 3.7.1. Effect on Na^+^/K^+^ ATPase

The level of Na^+^/K^+^ ATPase (0.6176 ± 0.1496) was significantly (*p* < 0.004) low in mice administered with ISO when compared to the mice in the control group (1.441 ± 0.1400). A significant increase in the levels of Na^+^/K^+^ ATPase was observed in mice treated with SFN, in both the prophylactic (1.087 ± 0.1221) and treatment groups (1.217 ± 0.2120) and also in the *α*-TCF- (1.473 ± 0.1366) treated group.

#### 3.7.2. Effect on Mg^2+^ ATPase

A significant (*p* < 0.001) increase in the level of Mg^2+^ ATPase (3.492 ± 0.3425) was observed in mice administered with ISO when compared to the mice in the control group (2.780 ± 0.2605). A significant decrease in the levels of Mg^2+^ ATPase was observed in mice treated with SFN, in both the prophylactic (2.470 ± 0.3532) and treatment groups (2.236 ± 0.09049) and also in the *α*-TCF- (1.825 ± 0.03871) treated group.

#### 3.7.3. Effect on Ca^2+^ ATPase

The level of Ca^2+^ ATPase (2.089 ± 0.2810) was significantly (*p* < 0.02) increased in mice administered with ISO when compared to the mice in the control group (1.257 ± 0.1339). A significant decrease in the levels of Ca^2+^ ATPase was observed in mice treated with SFN, in both the prophylactic (1.397 ± 0.1683) and treatment groups (1.239 ± 0.1588) and also in the *α*-TCF- (1.190 ± 0.2595) treated group.

### 3.8. Effect of Sulforaphane on Serum Biomarkers (Table 3)

#### 3.8.1. Effect on LDH

Mice administered with ISO showed increased levels of LDH (151.1 ± 11.42 IU/l), compared to the mice in the control group (53.97 ± 12.90 IU/l). A significant decrease in the levels of LDH was observed in mice treated with SFN, in both the prophylactic (42.63 ± 8.096 IU/l) and treatment groups (28.59 ± 10.78 IU/l) and also in the *α*-TCF- (63.57 ± 18.51 IU/l) treated group.

#### 3.8.2. Effect on CK

Mice administered with ISO showed increased levels of CK (122.7 ± 19.63 IU/l) compared to the mice in the control group (22.22 ± 3.036 IU/l). There was also a significant decrease in the levels of CK in mice treated with SFN, in both the prophylactic (67.93 ± 18.29 IU/l) and treatment groups (61.52 ± 21.25 IU/l) and also in the *α*-TCF- (58.88 ± 7.408 IU/l) treated group.

### 3.9. Effect of Sulforaphane on the Myocardium (Figure 3)

Histopathological observations of the heart tissues reveal clear cell membrane integrity without any cell inflammation in the control group (group 1). However, degenerative changes in the myocardial tissue, a smaller number of myocardial cells, and partial absence of the basement membrane were observed in mice administered with ISO. These changes indicated subendocardial necrosis of the heart tissue (group 2). The heart tissue of mice in both the prophylactic and treatment groups indicated mild necrosis with minimal inflammatory changes. A similar kind of protective effect towards mild necrosis with minimal inflammatory changes was observed in mice treated with *α*-TCF (group 5).

## 4. Discussion

ISO, a *β*-adrenergic agonist, produces severe oxidative stress in the heart, which causes infarct-like damage in the myocardium. Several mechanisms were proposed for ISO-induced myocardial damage, but the production of ROS in autoxidation of catecholamines is one of the most significant causal reasons [19]. This study showed that ISO-induced severe oxidative stress generates free radicals that stimulate lipid peroxidation and results in irreversible damage to the myocardial membrane. A similar effect was evidenced when a significant increase in the levels of lipid peroxidation was observed on performing the TBARS assay. TBARS, an oxidative marker, increases during the oxidative stress causing myocardial injury, which is most likely due to autoxidation of ISO [20, 21]. When ISO-administered mice are treated with SFN, a significant decrease in the TBARS concentration is observed. This indicates that SFN protects the heart from lipid peroxidation by removing the excess free radicals generated by ISO.

Endogenous antioxidant enzymes such as GSH, GPx, SOD, and CAT contain hydroxyl radical and superoxide anions, which play a prominent role in protecting the cell membranes against oxidative stress and also prevent ROS-induced cellular damage [22]. The presence of endogenous antioxidant enzymatic defense is critical for neutralizing ROS-mediated tissue injury. The primary free radical scavenging enzymes such as SOD, CAT, and GPx represent the first line of cellular defense against oxidative injury, decomposing oxygen (O_2_) and hydrogen peroxide (H2O2) before their interaction to form the more reactive hydroxyl radical [23]. SOD converts superoxide to hydrogen peroxide, and GPx and CAT are responsible for converting hydrogen peroxide to water. GSH directly reacts with free radicals or acts as an electron donor in the reduction of peroxides catalyzed by GPx [24].

When compared to the control group, the ISO-treated group showed decreased antioxidant enzyme levels. Other investigators observed similar changes in ISO-induced myocardial injury [20, 23, 25]. In this study, increased levels of GSH, GPx, SOD, and CAT, and restoration to normal levels in the SFN-treated groups indicated that SFN augmented the endogenous antioxidant enzymes in the mice myocardium.

Previous studies have found that enzymes such as LDH and CK-MB are present in the cardiac tissues and are used as a marker in cardiac tissue damage. Further investigations have also revealed that the levels of cardiac markers increased in ISO-treated subjects indicating that degenerative changes take place in cardiac tissues [22, 26]. When the myocardial cells containing LDH and CK are damaged due to altered myocardial cell metabolism and insufficient oxygen supply, this results in leakage of these enzymes into the bloodstream [19, 27, 28]. In this study, we observed that the level of serum markers decreased and was restored to the near-normal level in ISO-administered mice when treated with SFN, indicating the membrane stabilization effect of SFN in the mouse myocardium. ISO generates free radicals and enhances the lipid peroxidation of cardiac tissues leading mitochondrial swelling. As a result, mitochondrial enzymes such as Na^+^/K^+^, Mg^2+^2+, and Ca^2+^ ATPase are released causing an imbalance of myocardial electrolytes by significantly decreasing the Na^+^/k^+^ ATPase activity and increasing the Mg^2+^ and Ca^2+^ ATPase levels [19, 29–31]. The decreased activity of Na^+^/K^+^ ATPase decreases the rate of sodium efflux, thereby altering membrane permeability. Ca^2+^ ATPase is a key factor in regulating the activity of the calcium pump. Enhanced Ca^2+^ levels are observed in ISO-induced mice, which is due to the activation of adenylate cyclase by ISO. This overload of calcium in the myocardial cells during injury activates the Ca^2+^ ATPase of the membrane depleting high energy phosphate stores, thereby indirectly inhibiting Na^+^ and K^+^ transport and inactivation of Na^+^/K^+^ ATPase. The Mg^2+^ ATPase activity is involved in other energy-requiring processes in the cell, and its activity is sensitive to lipid peroxidation [19, 30, 31]. ISO-treated mice exhibited a decreased Na^+^/K^+^ ATPase activity and a significant increase in the Mg2+ ATPase and Ca^2+^ ATPase activities compared to the control group. A significant increase in the Na^+^/K^+^ ATPase activity and decrease in the Mg^2+^ and Ca^2+^ ATPase levels were observed in SFN-treated groups when compared to the ISO-treated group. These results suggest the protective effect of SFN against excessive oxidative damage of the myocardium by maintaining the membrane integrity through inhibition of lipid peroxidation in cell membranes. ISO-treated mice showed a decreased heart and body weight ratio, which might be due to reduced food and protein intake and inhibition of protein synthesis in cell necrosis or denaturation of proteins [32]. However, no significant difference was observed between the control and SFN-treated mice. A significant increase in the heart weight to body weight ratio was observed in the SFN-treated mice when compared with the ISO-treated mice. These results suggest that SFN may be protecting the heart from protein denaturation.

These biochemical reports were further supported by the histopathological studies. ISO-treated mice showed degenerative changes in the myocardium and showed a smaller number of myocardial cells and partial absence of the basement membrane and also necrosis of the cardiac tissue. This indicates a severe damage with higher magnification. SFN-treated mice showed a regenerative effect and reduced pathological changes, indicating the protective effect of SFN against ISO-induced myocardial injury.

## 5. Conclusion

The study suggest that SFN prevents ISO-induced myocardial injury by boosting the endogenous antioxidant activity, membrane stabilization, and restoring mitochondrial integrity. Besides, this study only confirmed the cardioprotective effects of pre- and posttreatment with SFN; therefore, further studies are required to confirm any long-term useful effects of SFN with specific molecular mechanism.

## Figures and Tables

**Figure 3:**
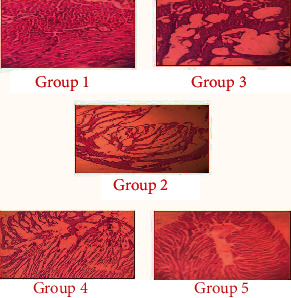
Light microscopy study of mouse myocardium treated with sulforaphane. Group 1: normal myocardial membrane integrity without any cell inflammation in the control group. Group 2: ISO-administered myocardial cells show degenerative changes and partial absence of the basement membrane. Groups 3 and 4: the heart tissue indicated the mild necrosis with minimal inflammatory changes (prophylactic and treatment group, respectively). Group 5: *α*-TCF-treated myocardium shows mild necrosis with minimal inflammatory changes.

**Table 1:** Effect of sulforaphane on oxidative stress and antioxidant biomarkers.

Treatment group	TBARS (nmol/g wet wt)	GSH (*μ*g/g wet wt)	GPx (*μ*g/g wet wt)	SOD (IU/mg protein)	CAT (IU/mg protein)
Group 1	55.01 ± 10.59	124.7 ± 2.442	17.41 ± 0.5711	39.13 ± 5.507	270.4 ± 8.789
Group 2	345.0 ± 41.81^a^	19.89 ± 1.702^a^	5.766 ± 0.4945^a^	13.70 ± 0.47^a^	87.95 ± 3.591^a^
Group 3	147.1 ± 16.3^b^	125.1 ± 2.175^b^	8.602 ± 0.3663^c^	55.69 ± 5.98^b^	308.5 ± 52.24^d^
Group 4	166.5 ± 11.5^b^	223.7 ± 1.635^b^	8.873 ± 0.4948^c^	57.00 ± 3.5^b^	419.8 ± 91.55^b^
Group 5	153.0 ± 5.61^b^	224.0 ± 2.216^b^	10.94 ± 0.7333^b^	58.53 ± 2.397^b^	284.5 ± 27.49^d^

All values are expressed as mean ± SEM. One-way ANOVA followed by Dunnett's posttest applied; ^a^*p* < 0.0001 vs. group 1, ^b^*p* < 0.0001 vs. group 2, ^c^*p* < 0.001 vs. group 2, and ^d^*p* < 0.05 vs. group 2.

**Table 2:** Effect of sulforaphane on mitochondrial ATPase enzymes.

Treatment group	Na^+^-K^+^ ATPase^1^	Mg^2+^ ATPase^1^	Ca^2+^ ATPase^1^
Group 1	1.441 ± 0.14	2.780 ± 0.26	1.257 ± 0.13
Group 2	0.6176 ± 0.15^a^	3.492 ± 0.34	2.089 ± 0.28^b^
Group 3	1.087 ± 0.12	2.470 ± 0.35^c^	1.397 ± 0.16±
Group 4	1.217 ± 0.21^c^	2.236 ± 0.09^d^	1.239 ± 0.16^c^
Group 5	1.473 ± 0.14^d^	1.825 ± 0.04^c^	1.190 ± 0.26^c^

All values are expressed as mean ± SEM. One-way ANOVA followed by Dunnett's posttest applied; ^a^*p* < 0.0001 vs. group 1, ^b^*p* < 0.05 vs. group 1, ^c^*p* < 0.05 vs. group 2, and ^d^*p* < 0.01 vs. group 2 (^1^moles of pi liberated/min/mg protein).

**Table 3:** Effect of sulforaphane on mitochondrial ATPase enzymes.

Treatment group	LDH (IU/l)	CK (IU/l)
Group 1	53.97 ± 12.90	22.22 ± 3.036
Group 2	151.1 ± 11.42^a^	122.7 ± 19.63^a^
Group 3	42.63 ± 8.096^b^	67.93 ± 18.29
Group 4	28.59 ± 10.78^b^	61.52 ± 21.25^c^
Group 5	63.57 ± 18.51^b^	58.88 ± 7.408^c^

All values are expressed as mean + SEM. One-way ANOVA followed by Dunnett's posttest applied; ^a^*p* < 0.0001 vs. group 1, ^b^*p* < 0.001 vs. group 2, and ^c^*p* < 0.05 vs. group 2.

## Data Availability

The data presented in this work are freely accessible to any other concerned researchers or students.
